# Design and Fabrication of α-MnO_2_-Nanorods-Modified Glassy-Carbon-Electrode-Based Serotonin Sensor

**DOI:** 10.3390/bios12100849

**Published:** 2022-10-09

**Authors:** Mohd Quasim Khan, Rais Ahmad Khan, Ali Alsalme, Khursheed Ahmad, Haekyoung Kim

**Affiliations:** 1 Department of Chemistry, M.M.D. College, Moradabad, M.J.P. Rohilkhand University, Bareilly 244001, UP, India; 2 Department of Chemistry, College of Science, King Saud University, Riyadh 11451, Saudi Arabia; 3 School of Materials Science and Engineering, Yeungnam University, Gyeongsan 38541, Korea

**Keywords:** α-MnO_2_/GCE, serotonin, sensor, electrochemistry

## Abstract

Serotonin is a very important monoamine neurotransmitter, which takes part in biological and psychological processes. In the present scenario, design and fabrication of a serotonin electrochemical sensor is of great significance. In this study, we have synthesized α-MnO_2_ via a hydrothermal synthesis method using potassium permanganate as a precursor. The physiochemical properties, such as structural and phase-purity of the prepared α-MnO_2_, were investigated by various characterization techniques and methods (powder X-ray diffraction, scanning electron microscopy, and energy-dispersive X-ray spectroscopy). Furthermore, the serotonin sensor was fabricated using α-MnO_2_ as an electrode modifier or electro-catalyst. The bare glassy carbon electrode (GCE) was adopted as a working substrate, and its active carbon surface was modified with the synthesized α-MnO_2_. This modified GCE (**α-MnO_2_/GCE = MGCE**) was explored as a serotonin sensor. The electrochemical investigations showed that the **MGCE** has excellent electro-catalytic properties towards determination of serotonin. The **MGCE** exhibits an excellent detection limit (DL) of 0.14 µM, along with good sensitivity of 2.41 µAµM^−1^ cm^−2^. The **MGCE** also demonstrated excellent selectivity for determination of serotonin in the presence of various electro-active/interfering molecules. The **MGCE** also exhibits good cyclic repeatability, stability, and storage stability.

## 1. Introduction

Serotonin, which is a monoamine neurotransmitter, plays a significant role in biological, physical, and psychological processes, such as appetite control, sleep pattern, sexual activity, and aggression [1,2]. The amount of serotonin in the human body should be controlled, and the normal range of serotonin in serum lies between 0.57 and 2.0 µM, whereas its normal amount in urine is considered in the range of 0.295–0.687 µM [3]. Serotonin has the potential to control and maintain all vital activities [4]. Presence of a low level of serotonin may cause various diseases, such as sexual dysfunction, Alzheimer’s disease, depression, and Parkinson’s disease [5,6]. Thus, it is of great significance to design and develop a highly precise, rapid, and sensitive technique or method for detection of serotonin level in human bio-fluids [7]. This can be helpful to diagnose diseases linked to serotonin. In previous years, various methods and techniques, such as high-performance liquid chromatography, capillary electrophoresis, enzyme immunoassay, fluorescence spectrophotometry, and mass spectrometry, were used as sensing platforms for determination of serotonin [8,9,10,11,12]. Although the above conventional methods were widely used for determination of serotonin, including other analytes, there are some limitations, such as complicated pretreatment processes, being time-consuming, and requiring expensive instruments, including well-trained operators [13]. These factors restrict rapid detection of serotonin with frequent testing, and it will be of great importance to discover or develop other alternative methods for rapid determination of serotonin [14]. Electrochemistry-based analytical or electrochemical methods have gained enormous attention because of their simple process, fast response/detection, ease of operation, and selectivity [15,16,17,18,19]. In addition, electrochemical techniques offer highly sensitive analysis with a cost-effective miniaturized platform [20]. Previously, a number of electrochemistry-based sensors were developed for sensing of serotonin. Indeed, various electrode materials, such as zinc oxide, nickel hydroxide (NiOH)_2_, carbon nanotubes, reduced graphene oxide, tungsten trioxide, gold nanoparticles, polymers, zirconium oxide (ZrO_2_), copper oxide, cobalt oxide, iron oxide, carbon nitride, and their hybrid composites, have been explored as serotonin sensing material [13,21,22,23,24,25,26,27,28]. However, there is still a need to develop a cost-effective and highly sensitive selective serotonin sensor.

Manganese oxide (α-MnO_2_) has gained enormous interest in the scientific community because of its excellent electro-catalytic properties, better inherent molecular adsorption ability, cost-effectiveness, excellent ion-exchange capability, natural abundance, and non-toxicity [29]. Further, α-MnO_2_ has been widely explored in a variety of applications, such as sensors, dye-sensitized solar cells, energy storage, batteries, catalysis, and dye degradation [30,31,32,33,34,35]. Previous reports showed that α-MnO_2_ is a potential candidate for fabrication of electrochemical sensors [30]. Herein, we have developed a serotonin sensor using α-MnO_2_ as electrode material. The α-MnO_2_-based serotonin sensor exhibits excellent performance. 

## 2. Materials and Methods

### 2.1. Chemicals

Potassium permanganate (KMnO_4_), serotonin, and hydrochloric acid (HCl, 37%) were purchased from Merck. Ascorbic acid and glucose were purchased from Sigma. Nafion was purchased from Sigma. Phosphate buffer saline solutions (PBS) were purchased from Loba. Uric acid was purchased from Alfa Aesar. Dopamine was purchased from TCI. Urea was purchased from Fischer Scientific. 

### 2.2. Synthesis of α-MnO_2_

The α-MnO_2_ was synthesized according to procedures reported elsewhere with some minor modifications [31]. In brief, 3 mmol of KMnO_4_ was dissolved in 25 mL of deionized (D.I.) water and 7.5 mmol of HCl was added. 

**Scheme 1:**
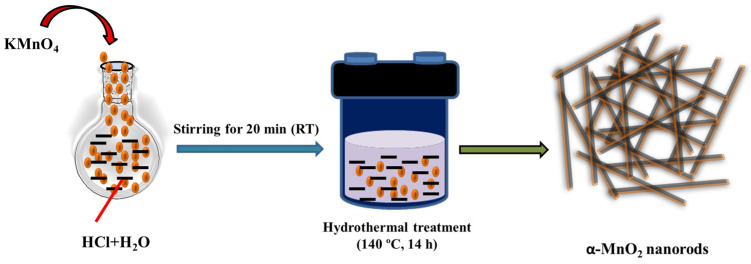
Pictorial representation for synthesis of α-MnO_2_.

This solution was stirred for 20 min at room temperature (RT), and transparent purple colored solution was obtained. This purple-colored solution was poured in to the Teflon-lined (capacity 50 mL) stainless steel autoclave reactor, which was heated at 140 °C for 12 h (Scheme 1). The precipitate was collected by centrifugation and washed with distilled water several times and dried at 70 °C overnight in vacuum oven. 

### 2.3. Instrumental Characterization

The phase-purity and confirmation of successful formation of α-MnO_2_ were authenticated by various characterization techniques. In this connection, powder X-ray diffraction (PXRD) pattern of the hydrothermally synthesized α-MnO_2_ was collected on Rigaku, Japan (RINT 2500 V XRD Instrument) with Cu Ka irradiation at two-Theta range of 10–80°. The structural phase of the prepared α-MnO_2_ was identified using PXRD investigations. The morphological characteristic of the prepared α-MnO_2_ was examined by utilizing scanning electron microscopy (SEM). The SEM images of the prepared α-MnO_2_ were collected on S-4800 (Hitachi) instrument. The elemental composition of the hydrothermally prepared α-MnO_2_ was investigated by utilizing energy-dispersive X-ray spectroscopy (EDS). The EDS spectrum of the hydrothermally prepared α-MnO_2_ was collected on Horiba EDS instrument. Electrochemical investigations were carried out using CH Instrument (silver/silver electrode was used as reference electrode, while platinum wire used as counter electrode). The bare glassy carbon electrode (BGCE) and α-MnO_2_-modified GCE (α-MnO_2/_GCE = **MGCE**) was used as working electrode. 

### 2.4. Fabrication of Working Electrode

**Scheme 2:**
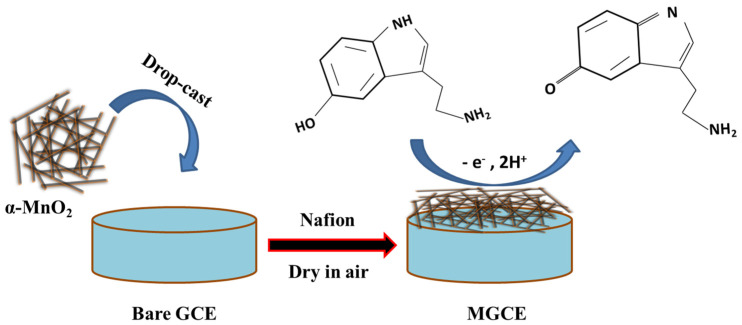
Pictorial representation for fabrication and working mechanism of **MGCE** towards serotonin sensing.

The α-MnO_2_ ink was prepared by dispersing 2.5 mg of α-MnO_2_ in 2 mL of distilled water (having 0.1% nafion) using ultrasonication for 30 min. Nafion was used as binder to increase the adhesiveness of the α-MnO_2_ on GCE surface. Further, 8.5 µL of the prepared ink was drop-casted on to the carbon surface of the GCE and dried in air for 4 h (Scheme 2). 

## 3. Results

### 3.1. Physiochemical Properties of α-MnO_2_

**Figure 1:**
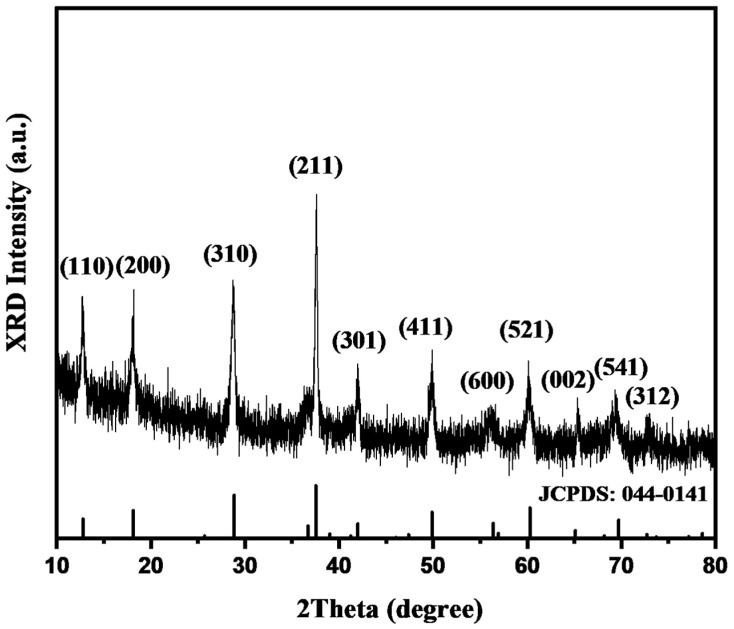
PXRD pattern of the hydrothermally prepared α-MnO_2_.

The collected PXRD data of the prepared α-MnO_2_ are presented in Figure 1. The PXRD pattern revealed the presence of several diffraction peaks at ~12.72, 18.13, 28.75, 37.70, 42.01, 49.96, 56.26, 60.12, 65.44, 69.41, and 72.96°. These diffraction peaks of 12.72, 18.13, 28.75, 37.70, 42.01, 49.96, 56.26, 60.12, 65.44, 69.41, and 72.96° can be assigned to the well-defined (110), (200), (310), (211), (301), (411), (600), (521), (002), (541), and (312) diffraction planes of α-MnO_2_. The obtained PXRD pattern of the hydrothermally synthesized α-MnO_2_ was found to be in good agreement with previous JCPDS card number 044-0141. The PXRD pattern of the prepared α-MnO_2_ does not show any other diffraction peak for impurity, which suggests successful formation of α-MnO_2_ with decent phase-purity. The crystallite size of the prepared α-MnO_2_ was determined by employing the Scherrer equation, as provided below:

(1) $$D=\frac{K\;\lambda }{\beta \mathrm{Cos}\;\theta }$$

(In the above equation 1, K = Scherrer constant, which is equal to 0.9, D is the average crystallite-size (Å), whereas λ is the X-ray wavelength (0.154 nm), and θ is the diffraction-angle; β represents full width at half-maximum (FWHM) of the observed peak). The crystallite size of the α-MnO_2_ was found to be ~31.4 nm using the Scherrer equation. Our obtained PXRD results for the prepared α-MnO_2_ are in good agreement with previously published literature and suggest successful formation of α-MnO_2_ with good phase-purity. 

**Figure 2:**
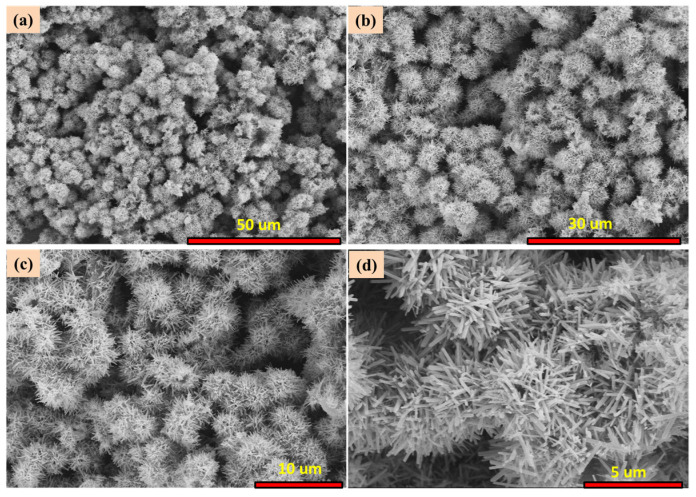
SEM pictures of the prepared α-MnO_2_ at different magnifications (**a**–**d**).

**Figure 3:**
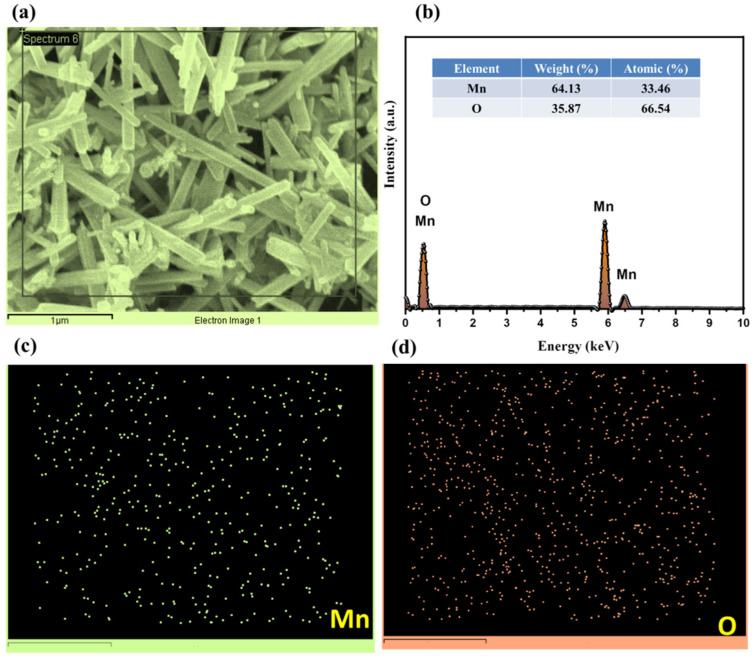
EDX electron image (**a**), EDX spectrum (**b**), and mapping images (**c**,**d**) of the prepared α-MnO_2_.

It has been determined from the reported studies that surface morphology of electrode materials plays a significant role and influences the electrochemical performance of the developed sensors. In this regard, it is necessary to investigate the morphological characteristics of the prepared α-MnO_2_ material. The obtained SEM pictures of the synthesized α-MnO_2_ at different magnifications have been displayed in Figure 2a–d. The SEM results indicate that hydrothermally prepared α-MnO_2_ comprised a rod-like surface. Therefore, it is clear that α-MnO_2_ has been successfully obtained with a rod-like surface morphology of a nanometer in size. To further verify the phase-purity of the hydrothermally prepared α-MnO_2_, it is necessary to examine the elemental composition of the hydrothermally prepared α-MnO_2_. Hence, we have examined the elemental composition of the hydrothermally prepared α-MnO_2_. The EDS results of the α-MnO_2_ are presented in Figure 3a–d. The EDS electron image of the hydrothermally prepared α-MnO_2_ is presented in Figure 3a, while the EDS spectrum of the α-MnO_2_ is depicted in Figure 3b. The EDS spectrum of the α-MnO_2_ exhibits the presence of Mn and O elements, which indicates that α-MnO_2_ has been successfully prepared. 

The EDS mapping image of the Mn and O elements has been displayed in Figure 3c and Figure 3d, respectively. The atomic percentage of the Mn and O elements was found to be 33.46 and 66.54%, whereas the weight percentage of the Mn and O elements was found to be 64.13 and 35.87%, respectively. 

No other element was observed in the EDS spectrum of the hydrothermally prepared α-MnO_2_. This suggests that α-MnO_2_ has good phase-purity. The above overall PXRD, SEM, and EDX results are consistent with previous reports and authenticated the formation of α-MnO_2_ with a nanorods-like surface morphology. According to the previous literature [31], electrode materials with rod-like surface morphology are the desirable materials for construction of electrochemical sensors. The rod-like surface of the electrode materials provides better bath and fast electron transportation. Thus, we have fabricated a serotonin electrochemical sensor using GCE as a working substrate and α-MnO_2_ as electrode material. 

### 3.2. Electrochemical Properties of MGCE

**Figure 4:**
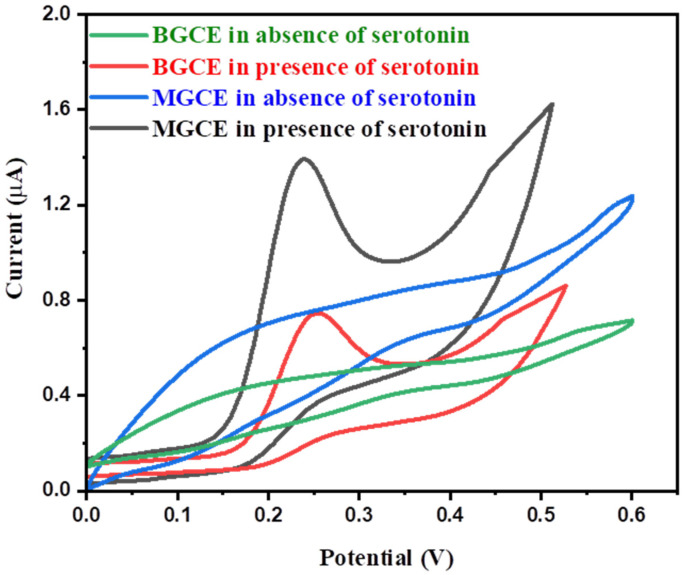
CVs of BGCE and **MGCE** in absence and presence of 2 µM serotonin in 0.1 M PBS (pH = 7.0) at scan rate of 50 mV/s.

The electrochemical activity of the BGCE and **MGCE** were determined using cyclic voltammetry (CV). The CVs of the BGCE and **MGCE** were taken in presence and absence of 2 µM serotonin in 0.1 M PBS of pH 7.0 at an applied scan rate of 50 mV/s. The collected CVs of the BGCE and **MGCE** in absence and presence of 2 µM serotonin in 0.1 M PBS of pH 7.0 at an applied scan rate of 50 mV/s are displayed in Figure 4. The BGCE showed poor electro-catalytic activity, whereas **MGCE** showed slightly enhanced electro-catalytic activity in absence of serotonin (Figure 4). The BGCE exhibited a current response of 0.77 µA for oxidation of 2 µM serotonin in 0.1 M PBS of pH 7.0 at an applied scan rate of 50 mV/s. Conversely, **MGCE** showed an improved current response of 1.41 µA for the oxidation of 2 µM serotonin in 0.1 M PBS of pH 7.0 at an applied scan rate of 50 mV/s (Figure 4). The CVs observations revealed that **MGCE** has higher current response compared to BGCE (Figure 4). This improved current response indicates successful deposition of α-MnO_2_ on GCE surface. In addition, the improved electro-catalytic activity of the **MGCE** can be attributed to the presence of good electrochemical features of α-MnO_2_. We selected **MGCE** as the serotonin sensor for further electrochemical investigations. 

**Figure 5:**
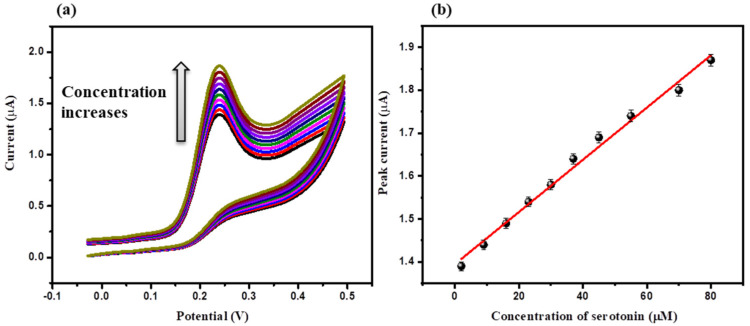
CVs (**a**) of **MGCE** in various concentrations (2 µM, 9 µM, 16 µM, 23 µM, 30 µM, 37 µM, 45 µM, 55 µM, 70 µM, and 80 µM) of serotonin in 0.1 M PBS (pH = 7.0) at fixed scan rate of 50 mV/s. Calibration curve (**b**) between peak current response versus concentration of serotonin.

The concentration of the analyte has the potential to affect the electrochemical performance of the fabricated electrochemical sensors. It has also been observed from the previous reports that the concentration of serotonin largely influences the electrochemical sensing performance of the constructed sensors. Thus, it is important to study the effect of concentration of serotonin on the electrochemical performance of the fabricated **MGCE**. Hence, we collected CVs of the **MGCE** in various concentrations of serotonin (2 µM, 9 µM, 16 µM, 23 µM, 30 µM, 37 µM, 45 µM, 55 µM, 70 µM, and 80 µM) in 0.1 M PBS (pH = 7.0) at an applied scan rate of 50 mV/s. The obtained CVs of the **MGCE** in various concentrations of serotonin (2 µM, 9 µM, 16 µM, 23 µM, 30 µM, 37 µM, 45 µM, 55 µM, 70 µM, and 80 µM) in 0.1 M PBS (pH = 7.0) at an applied scan rate of 50 mV/s are displayed in Figure 5a. 

The observations showed that current response of the **MGCE** increases with increasing the concentration of serotonin from 2 µM to 80 µM (Figure 5a). This indicated that concentration of serotonin has a significant role on electrochemical activity of **MGCE**. The calibration curve between the peak current response of **MGCE** and serotonin concentration were plotted to ascertain the linear relation. The calibration curve of the peak current response of the **MGCE** versus concentration of serotonin is presented in Figure 5b. This calibration curve suggests that current response increases linearly with increasing concentration of serotonin (Figure 5b), with R^2^ = 0.99. 

**Figure 6:**
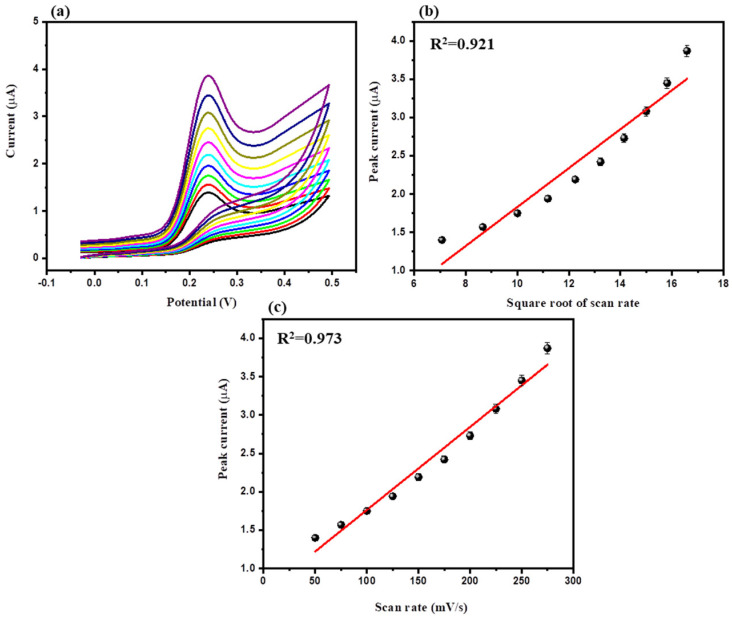
CVs (**a**) of **MGCE** in 2 µM serotonin in 0.1 M PBS (pH = 7.0) at various scan rates (50, 75, 100, 125, 150, 175, 200, 225, 250, and 275 mV/s). Calibration curve between peak current response versus square root of scan rate (**b**). Calibration curve between peak current response versus scan rate (**c**).

**Figure 7:**
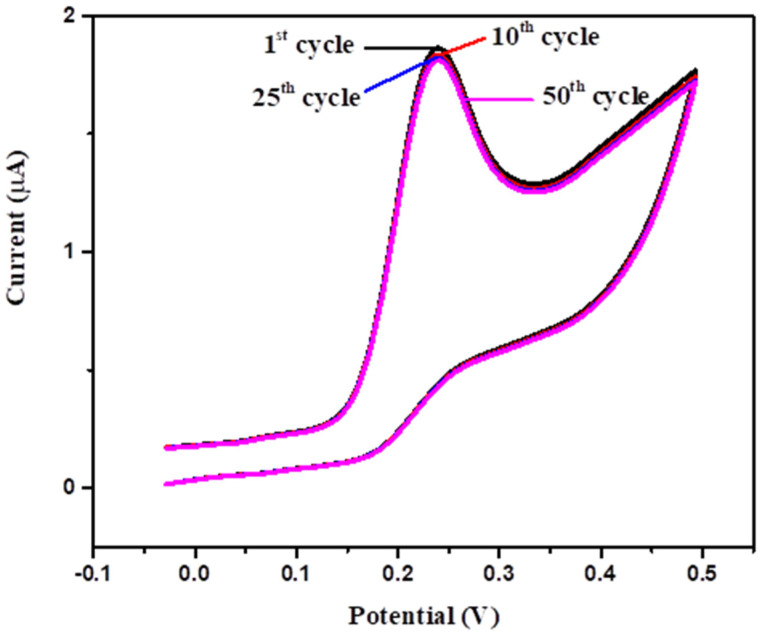
CVs of **MGCE** (1st, 10th, 25th, and 50th cycle) in 80 µM serotonin in 0.1 M PBS (pH = 7.0) at scan rate of 50 mV/s.

The applied scan rate also plays an important role in electrochemical sensing investigations. We also studied the effect of various applied scan rates on the electrochemical performance of the **MGCE** at a fixed concentration. The CVs of the **MGCE** were taken in the presence of 2 µM serotonin in 0.1 M PBS (pH = 7.0) at various applied scan rates (50, 75, 100, 125, 150, 175, 200, 225, 250, and 275 mV/s). The CVs results showed that the current response of the **MGCE** increases when the applied scan rate changes from 50 mV/s to 75 mV/s. Further investigations showed that current response increases while increasing the applied scan rate from 50 mV/s to 225 mV/s (Figure 6a). The calibration curve of the peak current response and square root of the applied scan rate is presented in Figure 6b, with an R^2^ value of 0.92. Further, we also plotted the calibration curve between the peak current response of the **MGCE** and applied scan rates. Figure 6c shows that the current response of the **MGCE** increases linearly while increasing the applied scan rate with R^2^ = 0.973. This suggests that detection of serotonin on an **MGCE** surface involves the adsorption process compared to the diffusions process. The cyclic stability and repeatability of the electrochemical sensors are the most important and desirable features for practical purposes. It is important to check the cyclic repeatability and stability of the fabricated **MGCE** for detection of serotonin. Thus, we collected 50 consecutive CVs of the **MGCE** in 80 µM serotonin in 0.1 M PBS (pH = 7.0) at a scan rate of 50 mV/s. The obtained CVs of the **MGCE** for serotonin detection are displayed in Figure 7. 

**Figure 8:**
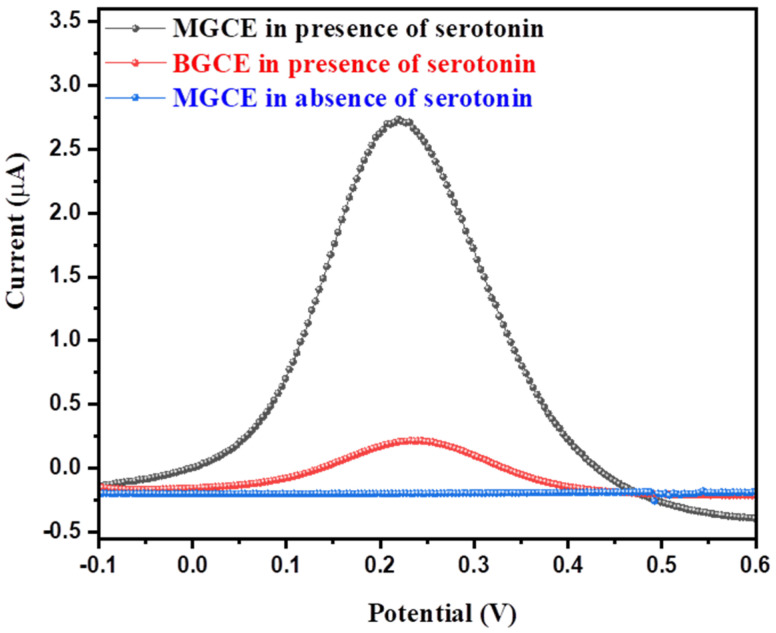
DPVs of BGCE and **MGCE** in absence and presence of 2 µM serotonin in 0.1 M PBS (pH = 7.0) at scan rate of 50 mV/s.

The CVs results showed insignificant variation in the current response of the **MGCE** after 50 consecutive cycles. The 1st, 10th, 25th, and 50th CV cycles of **MGCE** in 80 µM serotonin in 0.1 M PBS (pH = 7.0) at a scan rate of 50 mV/s have been displayed in Figure 7, which suggests excellent repeatability and cyclic stability of the **MGCE** up to 50 cycles, and it retained more than 91% of its initial performance in terms of current response. According to previous reports [13], it has been observed that the differential pulse voltammetry (DPV) method is a more effective and sensitive technique compared to CV. Thus, we have also obtained DPVs of BGCE and **MGCE** in the presence of 2 µM serotonin at an applied scan rate of 50 mV/s in 0.1 M PBS of pH 7.0. The obtained DPVs of the BGCE and **MGCE** are presented in Figure 8.

The BGCE shows a poor current response of 0.24 µA for detection of 2 µM serotonin in 0.1 M PBS (pH = 7.0) at a scan rate of 50 mV/s. The **MGCE** demonstrated an enhanced current response of 2.75 µA for detection of 2 µM serotonin in 0.1 M PBS (pH = 7.0) at a scan rate of 50 mV/s. Thus, **MGCE** has excellent electro-catalytic properties compared to BGCE, which may be due to the presence of α-MnO_2_ in the fabricated **MGCE**. The DPVs investigations showed better electrochemical performance for **MGCE** compared to the CVs. The DPV current response of the **MGCE** was also recorded in absence of serotonin, and the obtained results did not show any significant response (Figure 8). Thus, we have selected **MGCE** as the working electrode and DPV as an efficient detection technique for further electrochemical sensing studies. The above CVs showed that concentration of serotonin influences the electrochemical performance of **MGCE**. Thus, we have studied the effect of various concentrations (2 µM, 6 µM, 10 µM, 14 µM, 18 µM, 22 µM, 26 µM, 30 µM, 35 µM, 40 µM, 45 µM, 50 µM, 55 µM, 60 µM, 65 µM, and 70 µM) of serotonin on the electrochemical activity of **MGCE** (0.1 M PBS of pH 7.0 (applied scan rate = 50 mV/s). 

**Figure 9:**
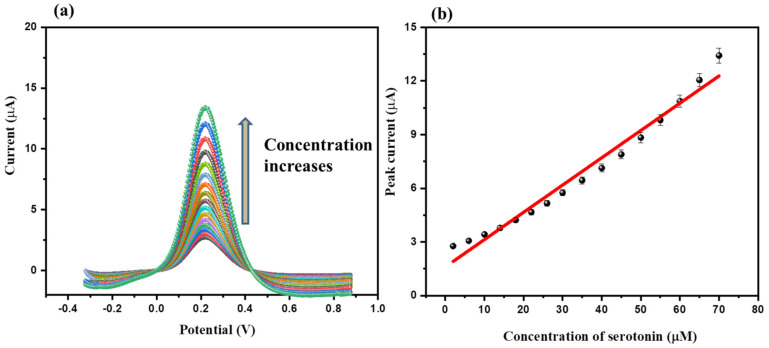
DPVs (**a**) of **MGCE** in various concentrations (2 µM, 6 µM, 10 µM, 14 µM, 18 µM, 22 µM, 26 µM, 30 µM, 35 µM, 40 µM, 45 µM, 50 µM, 55 µM, 60 µM, 65 µM, and 70 µM) of serotonin in 0.1 M PBS (pH = 7.0) at scan rate of 50 mV/s. Calibration plot (**b**) between current response versus concentration of serotonin.

The obtained DPVs of the **MGCE** in different concentrations of serotonin (2 µM, 6 µM, 10 µM, 14 µM, 18 µM, 22 µM, 26 µM, 30 µM, 35 µM, 40 µM, 45 µM, 50 µM, 55 µM, 60 µM, 65 µM, and 70 µM) at a fixed scan rate of 50 mV/s (0.1 M PBS; PBS = 7.0) are summarized in Figure 9a. The DPVs show that current response increases with respect to concentration of serotonin. The calibration curve between current response versus concentration of serotonin is shown in Figure 9b. The polynomial fitted calibration curve between peak current response versus concentration of serotonin is presented in Figure S1. 

**Figure 10:**
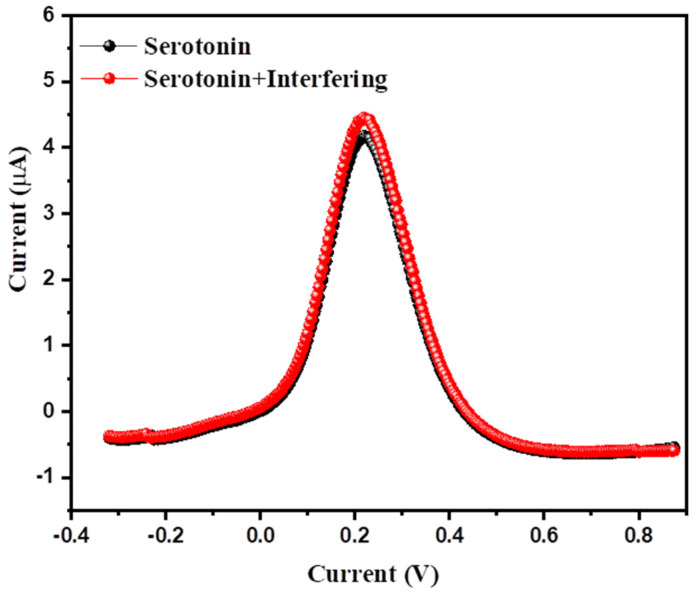
DPVs of **MGCE** in 20 µM of serotonin and serotonin + interfering species (urea, glucose, dopamine, hydroquinone, ascorbic acid) in 0.1 M PBS (pH = 7.0) at scan rate of 50 mV/s.

Selectivity of the electrochemical sensors is one of the most important desirable features for practical purposes. Thus, it is of great importance to examine the selectivity of the fabricated **MGCE**. The DPV graph of the **MGCE** was obtained in the presence of 20 µM serotonin, and 20 µM serotonin + interfering species (such as urea, hydroquinone, glucose, dopamine, ascorbic acid, etc.) were collected at a fixed scan rate of 50 mV/s (Figure 10). The concentration of interfering species was five times higher than that of the serotonin. All the interfering species were mixed with the 20 µM serotonin solution. The observations suggest that the presence of interfering species could not significantly influence the electrochemical performance of **MGCE**. This indicates that **MGCE** has excellent selectivity for serotonin (Figure 10). The concentration of interfering species was higher than that of serotonin. 

**Figure 11:**
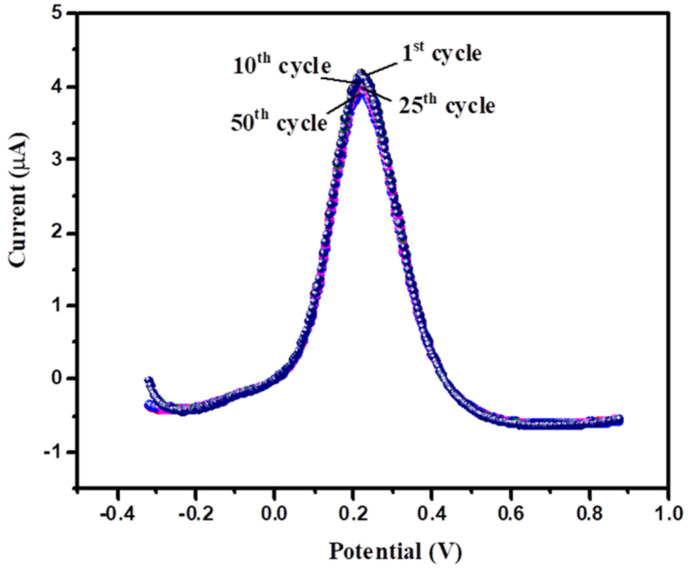
1st, 10th, 25th, and 50th DPVs of **MGCE** in 20 µM of serotonin in 0.1 M PBS (pH = 7.0) at scan rate of 50 mV/s.

Repeatability of the electrochemical sensor is another significant desirable characteristic. The repeatability of the **MGCE** was also investigated using DPV. The 50 consecutive DPV graphs of the **MGCE** were obtained in the presence of 20 µM serotonin at a scan rate of 50 mV/s. The 1st, 10th, 25th, and 50th DPV graphs of the **MGCE** in the presence of 20 µM serotonin in 0.1 M PBS (pH = 7.0) at a scan rate of 50 mV/s are presented in Figure 11. 

There was insignificant variation/change observed, which suggests good repeatability and stability up to 50 cycles and revealed that **MGCE** retained ~89.7% of its initial performance in terms of current response. Reproducibility of the **MGCE** was also examined by fabricating four different **MGCE**s. The DPVs of the four freshly prepared **MGCEs** were obtained in 20 µM of serotonin in 0.1 M PBS (pH = 7.0) at a scan rate of 50 mV/s. The obtained results are depicted in Figure S2 and showed good reproducibility. 

**Figure 12:**
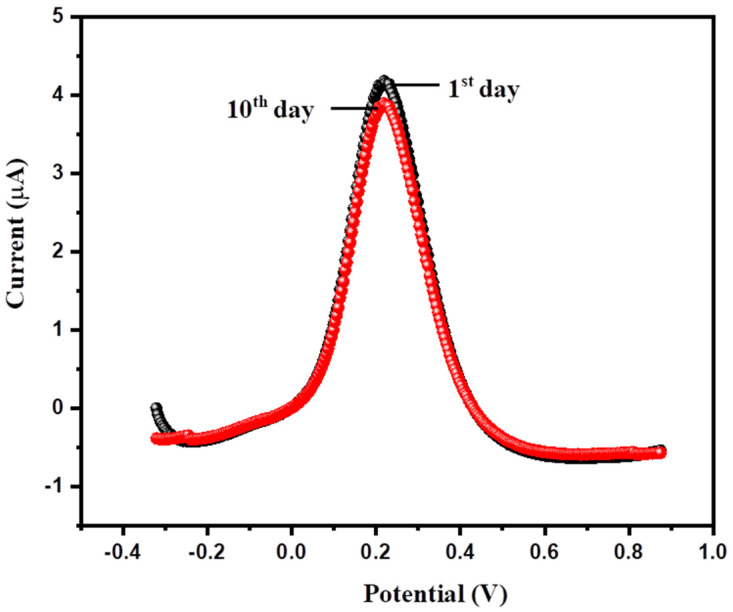
DPVs of **MGCE** (1st day and after 10th day) in 20 µM of serotonin in 0.1 M PBS (pH = 7.0) at scan rate of 50 mV/s.

The storage stability of the **MGCE** was also checked using DPV. The **MGCE** was stored for 10 days in a vacuum desiccator, and the DPV curve of the **MGCE** was recorded in the presence of 20 µM serotonin in 0.1 M PBS of pH 7.0 at a scan rate of 50 mV/s, and the obtained results are compiled in Figure 12. 

The obtained results suggest good storage stability after 10 days and retained more than 84% of the initial performance in terms of current response. 

Real sample analysis was also conducted using the standard addition method. The 20 µM of serotonin was added to the urine sample (collected from healthy person, male, age 29 years old), and the DPV curve was recorded as shown in Figure S3. The observations showed a good recovery of 98.4% using the standard addition method. Furthermore, we also investigated the selectivity of the **MGCE** in a urine sample. The DPV of the **MGCE** was recorded in the presence of 20 µM serotonin + glucose, 20 µM serotonin + urea, 20 µM serotonin + ascorbic acid, 20 µM serotonin + hydroquinone, and 20 µM serotonin + dopamine at a scan rate of 50 mV/s (Figure S4). The concentration of interfering species was five times higher than that of serotonin. There was no significant change observed, which indicated good selective nature of **MGCE** for serotonin determination. 

The probable mechanism for detection of serotonin has been illustrated in Scheme 2. 

The electrochemical performance of the **MGCE** was evaluated by calculating detection limit (LoD) and sensitivity. The LoD and sensitivity of the **MGCE** were calculated using the following equations provided below:

(2) $$\mathrm{LoD}=\frac{3.3*\;\sigma b\;}{S}$$

(Herein, σ_b_ = standard deviation or error of the blank, and S = slope of the calibration curve).

(3) $$\mathrm{Sensitivity}=\frac{S}{A}$$

(where A = area of the electrode) 

The **MGCE** exhibited an excellent LoD and sensitivity of 0.14 µM and 2.41 µA µM^−1^cm^−2^, respectively. 

Over the past few years, various serotonin sensors were reported using nanostructured electrode materials. In this regard, Matt et al. [13] fabricated a ZrO_2_-based serotonin sensor that showed a good LoD of 0.585 µM. In other work, Babaei et al. [25] demonstrated the sensing behavior of Nafion/(Ni(OH)_2_/multi-walled carbon nanotubes (MWNTs)/GCE towards detection of serotonin and obtained an LoD of 0.083 µM. Rand et al. [26] employed carbon nanofibers (CNFs) as a serotonin sensor and the authors reported an LoD of 0.25 µM. In another report, Matuschek et al. [36] used a 3D mesoporous ITO electrode as a working electrode for detection of serotonin. This applied sensor exhibited an LoD of 7.5 µM using the DPV method. Reddaiah et al. [37] designed and fabricated poly-Alizarin Red S/MWCNTs as electrode material for construction of a serotonin sensor. This prepared material (poly-Alizarin Red S/MWCNTs) was deposited on GCE, which demonstrated an excellent LoD of 0.18 µM. Gupta et al. [38] also developed a serotonin sensor using novel strategies. The authors prepared polymelamine/pyrolytic graphite using a benign approach, and the working surface of GCE was modified with polymelamine/pyrolytic graphite as an electrode modifier. This fabricated electrode (polymelamine/pyrolytic graphite/GCE) exhibited a good LoD of 0.49 µM for sensing of serotonin [38]. Citicoline-sodium-modified carbon paste electrode (CDP-Choline/MCPE) was also constructed by Deepa et al. [39] and employed as a serotonin sensor. This sensor showed an LoD of 5.81 µM. In other work, Cernat et al. [40] fabricated a serotonin sensor using AuNPs@PPy/GSPE as a working electrode. This working electrode (AuNPs@PPy/GSPE)-based serotonin sensor exhibits an LoD of 32.22 µM [40]. Mahanthesh et al. [41] also utilized a graphite pencil as a working electrode and employed a serotonin sensor that showed a decent LoD of 4 µM [41]. Li et al. [42] prepared poly (basic red 9)-doped functionalized multi-walled carbon nanotubes and constructed a serotonin sensor. This serotonin sensor showed a good LoD of 9 µM using poly (basic red 9)-doped functionalized multi-walled carbon nanotubes as an electrode modifier [42]. In 2019, Shahid et al. [43] also fabricated a serotonin sensor using reduced graphene oxide/cobalt oxide (rGO/Co_3_O_4_) as an electrode-modifier. The authors fabricated GCE/rGO/Co_3_O_4_ as a serotonin sensor, which displayed an LoD of 1.1 µM. Another report based on nickel oxide/barium titanate (NiO/BaTiO_3_) also showed good performance for detection of serotonin [44]. Our obtained results for **MGCE** are comparable with previously reported sensors in terms of LoD, as listed in Table 1 [13,25,26,36,37,38,39,40,41,42,43,44].

## 4. Conclusions

In summary, we can conclude that nanorods of α-MnO_2_ have been obtained using a hydrothermal method, which was characterized by various advanced characterization methods. Further, a glassy-carbon-electrode-based cost-effective serotonin sensor was developed. The developed serotonin sensor (α-MnO_2_/GCE) showed excellent electrochemical performance for determination of serotonin in terms of sensitivity and detection limit using differential pulse voltammetry. In further investigations, α-MnO_2_/GCE also showed good repeatability, stability, and selectivity towards detection of serotonin. 

## Figures and Tables

**Table 1:** Comparison of LoD and linear range of **MGCE** with previous reports [13,25,26,36,37,38,39,40,41,42,43,44].

Material	LoD (µM)	Linear Range (µM)	References
MGCE	0.14	2–80	Present study
ZrO_2_	0.585	10–50	13
Nafion/Ni(OH)_2_/MWNTs/GCE	0.083	0.15–14.2	25
Carbon nanofibers	0.25	1–10	26
3D mesoporous ITO electrode	7.5	50–1000	36
Poly-Alizarin Red S/MWCNTs/GCE	0.18	0.5–10	37
Polymelamine/pyrolytic graphite/GCE	0.49	1–100	38
Citicoline-sodium-modified carbon paste electrode	5.81	10–30	39
AuNPs@PPy/GSPE	32.22	0.1–30	40
GPE	4	40–750	41
F-MWCNTs/BR9	9	10–83	42
rGO/Co_3_O_4_	1.1	1–10	43
NiO/BaTiO_3_	0.03	0.05–5	44

## Data Availability

Not applicable.

## Supplementary Materials

The following supporting information can be downloaded at: https://www.mdpi.com/article/10.3390/bios12100849/s1, Figure S1. Calibration curve between current response versus concentration of serotonin, Figure S2. DPVs of four freshly fabricated **MGCE** in 20 μM of serotonin in 0.1 M PBS (pH=7.0) at scan rate of 50 mV/s, Figure S3. DPV of **MGCE** in 20 μM of serotonin in urine at scan rate of 50 mV/s. Inset shows urine sample, Figure S4. DPVs of **MGCE** in 20 μM of serotonin with different interfering species in urine at scan rate of 50 mV/s.
