# Supplementary material for: Design and Fabrication of α-MnO2-Nanorods-Modified Glassy-Carbon-Electrode-Based Serotonin Sensor
Source: Biosensors (Basel). 2022 Oct 9;12(10):849. doi: 10.3390/bios12100849 (PMC9599580; doi:10.3390/bios12100849)
Supplement: Supplementary file 1 [file biosensors-12-00849-s001.zip › biosensors-1931624-supplementary.pdf]

# Supporting Information

# Design and Fabrication of $\alpha$-$MnO_2$-Nanorods-Modified Glassy-Carbon-Electrode-Based Serotonin Sensor

**Mohd Quasim Khan [1,†], Rais Ahmad Khan [2], Ali Alsalme [2,*], Khursheed Ahmad [3,†] and Haekyoung Kim [3,*]**

[1] Department of Chemistry, M.M.D. College, Moradabad, M.J.P. Rohilkhand University, Bareilly 244001, UP, India
[2] Department of Chemistry, College of Science, King Saud University, Riyadh 11451, Saudi Arabia
[3] School of Materials Science and Engineering, Yeungnam University, Gyeongsan 38541, Korea
* Correspondence: aalsalme@ksu.edu.sa (A.A.); hkkim@ynu.ac.kr (H.K.)
† These authors contributed equally to this work.

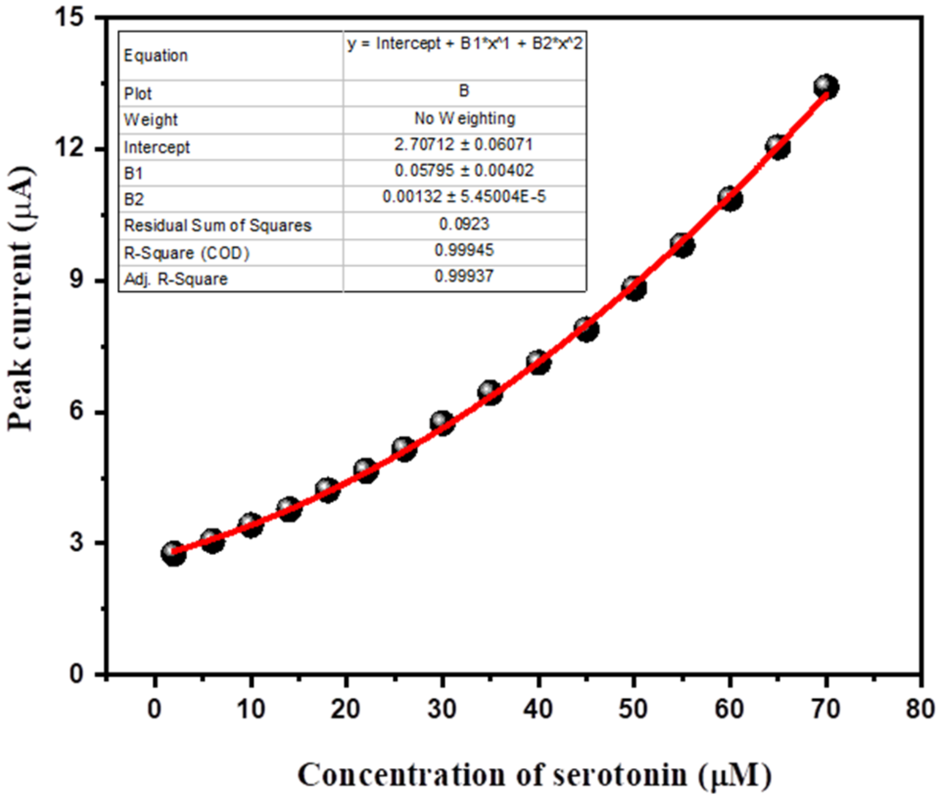


**Figure S1**. Calibration curve between current response versus concentration of serotonin.

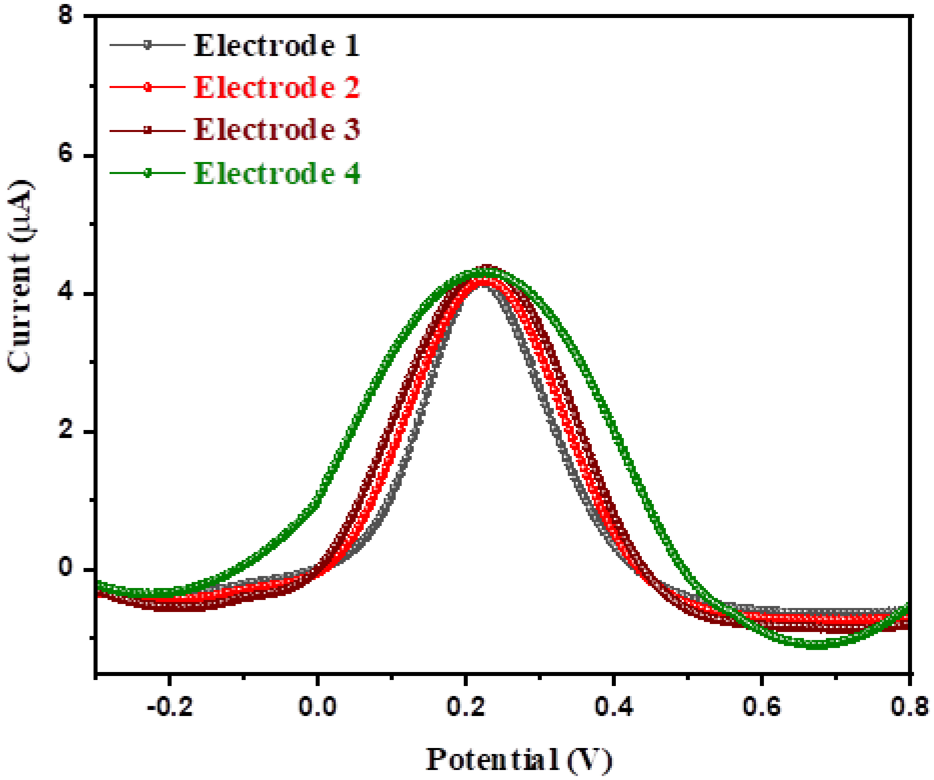


**Figure S2**. DPVs of four freshly fabricated MGCE in 20 µM of serotonin in 0.1 M PBS (pH=7.0) at scan rate of 50 mV/s.

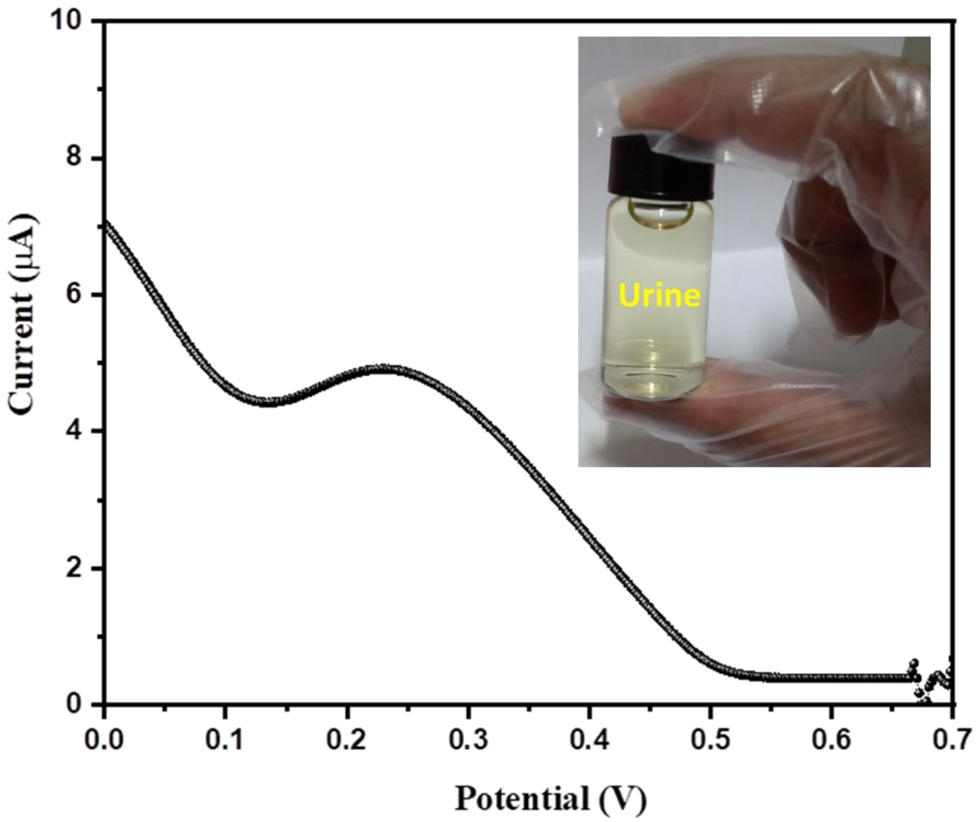


**Figure S3.** DPV of MGCE in 20 μM of serotonin in urine at scan rate of 50 mV/s. Inset shows urine sample.

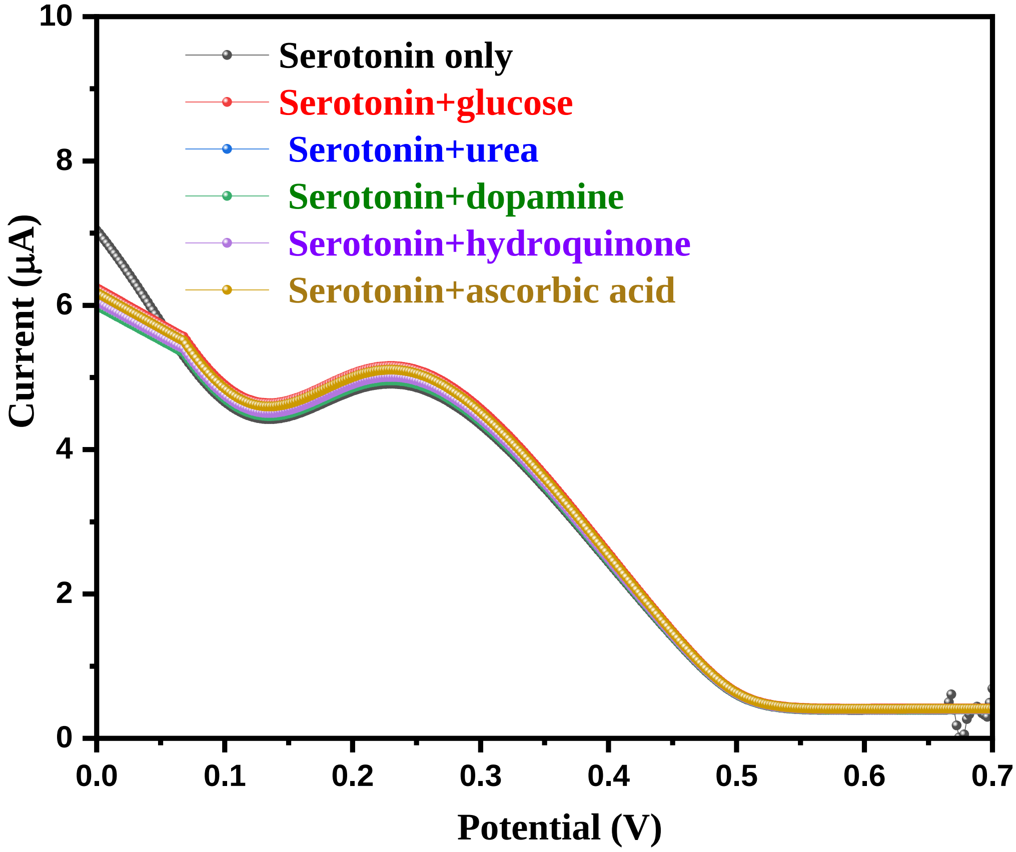


**Figure S4**. DPVs of MGCE in 20 µM of serotonin with different interfering species in urine at scan rate of 50 mV/s.
